# Supplementary material for: Detection of spike protein in term placentas of COVID-19 vaccinated and/or SARS-CoV-2 infected women
Source: PLoS One. 2026 Mar 5;21(3):e0344185. doi: 10.1371/journal.pone.0344185 (PMC12962466; doi:10.1371/journal.pone.0344185)
Supplement: Attachment — Submitted filename: Response to Reviewers.docx [file pone.0344185.s004.docx]

Dear Editor,

We are pleased to resubmit our revised manuscript entitled

**“Detection of Spike Protein in Term Placentas of COVID-19–Vaccinated and/or SARS-CoV-2–Infected Women,”**

which has been substantially improved.

Following the suggestion of Reviewer 1, we performed double immunostaining with anti-CD68 and anti-spike antibodies, both of which stained Hofbauer cells. Accordingly, we have included an additional figure to show the double staining.

All comments and suggestions from Reviewer 1 have been fully addressed. We have marked our answers and comments in purple. The manuscript has been proofread by a certified translator.

We hope that the revised manuscript now meets all criteria for publication in *PLOS ONE*.

Thank you very much for your time and consideration.

Kind regards,

Catharina Bartmann
on behalf of all authors

Reviewer #1: In this manuscript, the authors describe a cohort of pregnant people who experienced COVID-19 infection during the SARS-CoV-2 global pandemic who had received vaccination (or no vaccination) in Germany. The analysis includes detailed histological examination of placental tissues for various proteins associated with SARS-CoV-2. The authors describe their findings systematically, and test whether obstetric and neonatal outcomes were affected by SARS-CoV-2 infection or vaccination.

**Thank you.**

A major pitfall of the current investigation is the lack of co-staining with markers of Hofbauer cells for example to show localization of SARS-CoV-2-related proteins. I would say that this is necessary for publication.

**Thank you. We have addressed the major pitfall. CD68 is a well-established and widely used marker for the identification of Hofbauer cells. Therefore, we have performed double immunostaining with anti-CD68 and anti-spike antibodies, both of which stained Hofbauer cells. Accordingly, we have included an additional figure (new Figure 5, previous Figure 5 is now Figure 6) to further illustrate our findings.**

There are many instances of grammatical errors, spelling mistakes, and general misuse of punctuation that requires immediate correction. The authors are encouraged to more finely review the manuscript before initial submission to facilitate efficient review by reviewers. This manuscript reads as though the authors were not careful in their preparation of this manuscript for submission to PLOS ONE.

**Thank you for pointing this out.** **We have read the text carefully. Further, the manuscript has been proofread and corrected by a certified translator.**

Abstract:
• The abstract is well-written and concise. No amendments are recommended.
**Ok.**

Introduction:
• Lines 79-81: This sentence does not make grammatical sense. **Corrected**
The authors are encouraged to review the manuscript carefully for grammatical errors and inconsistencies. **Done**

• Line 83: Be careful with use of tenses. For example, here it should be “neonatal morbidities were reported in 2021”. There are multiple instances in this manuscript where past-tense should be used and not present-tense. **Done**
• Line 105: The comma is not needed here. **Done**
• Line 116: Comma after “we were interested” is not necessary, please remove. **Done**

Methods:
• Line 130: October not Oktober. **Done**
• Table 1: 2nd and 3rd spelled incorrectly. **Done**
• Line 150: Should be Women (plural), not Woman (singular). **Done**
• Please include dilutions used of all antibodies (perhaps in a table in the supplementary data).

**We have created an additional table (new Table 3) that contains the primary antibodies for immunohistochemistry, including the dilutions.**

Results:
• Starting at Line 227: Could any of these numbers be expressed as percentages so the reader can appreciate the proportions affected? **Done**
• Figures: Please capitalize the start of every individual text label (i.e., “Number of Patients”, “Symptoms” – both axis labels. **Done**
• Line 270: Correct to leukocytes not leucocytes. D**one**
• Table 4: Correct to negative, not negativ **Done**;

- Please clean up the figure and make sure things are capitalized where needed, and that abbreviations are listed in the footnotes. There are many instances of spelling mistakes that need to be corrected. **Thank you very much. We have improved the previous Figure 5 (now Figure 6).**
- Line 278: “was” not “war”. **Corrected**

• Line 281: hints? Should this be “hits”? **No, we mean hint in the meaning of “point to”. Now we have used the term “proof” since the RNA-detection indeed is a proof of the presence of the RNA in question, we have rewritten this sentence.**

• Line 283: what is “trimenon”? **trimenon is trimester, we have replaced it now with trimester**

• Figures 3&4:

Please add scale bars to your images. **Done.**

Further, if you are going to claim SARS-CoV-2 proteins in Hofbauer cells for example, you would absolutely need co-staining with a marker for Hofbauer cells like CD68 or CD163. I would say that without some distinct IHC staining of differing cell types this manuscript loses a lot of its strength. I would request co-staining likely by IF would be sufficient. I likely would not accept a paper for publication without this confirmation.
**Thank you for the comment. We have performed double staining and added a typical example as a new Figure 5.**

Discussion:
• Line 291: What is meant by “assumed emergency situation”? A conservative estimate of COVID-19 deaths globally puts it at 7 million deaths. More accurate estimates by the WHO the Lancet put the deaths at 16-18 million globally. If this isn’t an emergency, I don’t know what is.

**Well that’s still a matter of debate since overall mortality was not detected in the national statistics databases for Germany (** [**https://www.destatis.de/DE/Themen/Gesellschaft-Umwelt/Bevoelkerung/Sterbefaelle-Lebenserwartung/_inhalt.html#sprg229094**](https://www.destatis.de/DE/Themen/Gesellschaft-Umwelt/Bevoelkerung/Sterbefaelle-Lebenserwartung/_inhalt.html#sprg229094)**). Since our study groups were recruited in Würzburg/Germany, we refer to those data in our interpretation. According to a very new publication (Kuhbandner C, Reitzner M. 2025 Regional patterns of excess mortality in Germany during the COVID-19 pandemic: a state-level analysis. R. Soc. Open Sci. 12: 250790. https://doi.org/10.1098/rsos.250790**[**rsos.250790.pdf**](https://watermark02.silverchair.com/rsos.250790.pdf?token=AQECAHi208BE49Ooan9kkhW_Ercy7Dm3ZL_9Cf3qfKAc485ysgAAA0YwggNCBgkqhkiG9w0BBwagggMzMIIDLwIBADCCAygGCSqGSIb3DQEHATAeBglghkgBZQMEAS4wEQQMdq1tpjhHy04tcZaLAgEQgIIC-ZdRvG-7ehOiuRewd0AGtldgBvic-BLlGIN9OrilSleyBhioByuz2K38VKymW8AOhhNXSrfbrI5UVqQF9t5_JzAXWdGsilQJ13pjdZI9Sb5-QsojhbgSOSLqnx59azEPUvfyDcrUgmhq_W4PUSkPNjBL_6if3kyfsFG5vDQHJ25_AfD0cpkdW_aG95qP2fBwU8lXiGUexCDBmAPSgI5QHbkGzG90FEwZdDoThDwBbpE9WPq5t5c6y-24wyRtDG4akYoVCG65dbFJ-5ME_4knqr87nMZ_AN2DXpLOt_Vu02irxQwUWKchG8NkmoaRWE214wJvc8UlKx8nGpaC0KNNL-RCVak0Ye60jIiJb4qa8fSqdOZolUnP2KkzcCDLRrhut_ofOTknRN84Cq9ig5vIc-cqz0C2PVqvud0o76v1kj8NF4-XNPys92ZpdYFuh1d-UU1q4WAFsTI4GwTUT07s08ZILv03eMsXbFQC_Ubh-VE6qYLdhmRe9cMRvYD_ZblEoKxv3_LPdLlvInQEytUC1O3-XDoSPwnj5TIbdWgulYw93NnZWIOFl50hbQnBV5NTfz21oQG6xlfvWqFe13bZ8gy-1tC05QUlg_eGKWVcc7tHse7T0XH8eAY1sxifaxbgGvjvc-fbXf6rO1WOd0BDlBD0hwPS3Zuy44csanCyxfO3EuRyKZiRzVy0iBqMRKIhqMQu45w0_7tCapFoL2HNLi1qyrYktmpHOgPaMPU_9d1qSsyX2ySYDxfxIHapC3mrDdlk7UUZmghuVOJVm14A3ZgQQ9lixipur0X4PZmotWd4-GOBCOZVWK7gt16Na4rxZCYcdwFU5WAcNL7EbXeLqS6E_3KEE6iCQ8DIsAPGMIe4OKBWS-IubBVEvNRvsFgpS5yzOOozoCzL8jqq0ZRYB7910MFlVNaHURrCxryb4CbJWhoDpjotTSFJBnjub4v5Ku0ORfGfQ__4DZRbKTHa6yAzYRqUywL4a02ARUEplZFM1Jlho_2fdorf)**) there was only a moderate excess mortality – comparable to years with a severe influenza season, such as the flu season 2017/2018. However, we have deleted the phrase and replaced it by “following European guidelines”, to avoid this discussion, which is not a matter of our publication.**

• Line 300-301: This sentence is not understandable.

**We have improved the sentence adding “group compared to the general German population”.**

• **You have listed a major limitation of your study to exclude those with complicated births, and I would like to know why there was a conscious decision to include cases where COVID-19 may have caused harm to mother and fetus/neonate**.

**All women were asked during the recruitment period whether they would like to participate in the study. We did not exclude any premature births. However, we always ensured that the medical safety of the women and their children took priority. Overall, only a few women with premature births participated in the study, meaning that the average premature birth rate in Germany is underrepresented. The sentence “Women with miscarriages and early premature births were not part of the study population.” is misunderstanding, therefore we have improved it.**

• Line 349: What is trasplacentar? **This was a typo, it should mean “transplacental”. We have corrected this.**

Reviewer #2: Dear Authors:
This study investigated whether SARS-CoV-2 spike protein or vaccine-derived mRNA can be detected in term placentas. The cohort included 106 women who delivered at the University Hospital of Würzburg between November 2020 and October 2022. The strengths include:
1. Novelty: First reported detection of vaccine-derived mRNA traces in placental tissue in vivo.
2. Comprehensive Approach: Combined immunohistochemistry and RNAscope, increasing reliability of detection.

In general, this is a well-written manuscript, and I have no further comments.

**Thank you.**
