# Supplementary material for: Detection of spike protein in term placentas of COVID-19 vaccinated and/or SARS-CoV-2 infected women
Source: PLoS One. 2026 Mar 5;21(3):e0344185. doi: 10.1371/journal.pone.0344185 (PMC12962466; doi:10.1371/journal.pone.0344185)
Supplement: Attachment — Submitted filename: Response to Reviewers_R2_260212.docx [file pone.0344185.s005.docx]

**Response to Reviewers**

Reviewer #3:

After reviewing the manuscript, I find that it presents original observational research addressing a timely and sensitive topic the detection of SARS-CoV-2 spike protein and vaccine-related RNA signals in term placental tissue following maternal infection and/or vaccination. The study is based on a well-defined clinical cohort and applies appropriate histological and molecular techniques to address its descriptive aims.

Thank you!

From a technical standpoint, the experiments are conducted to an adequate standard and are described in sufficient detail to allow reproducibility. The immunohistochemical analyses are supported by appropriate controls, and the addition of double staining to identify Hofbauer cells strengthens the interpretation regarding cellular localization. The exploratory use of RNAscope provides an additional layer of evidence, which is presented as supportive rather than definitive.
The statistical analyses are appropriate for the study design, and the conclusions are generally aligned with the data. Ethical approval, informed consent, and data availability are clearly documented and meet the journal’s requirements.

Thank you!

Given the sensitive nature of the topic, I would encourage the authors to add a small amount of additional interpretative caution in the discussion. In particular, it would be useful to state explicitly that immunohistochemical detection of spike protein does not distinguish between locally produced protein and protein that has been taken up from the circulation, and that in macrophage-lineage cells such as Hofbauer cells the signal may also reflect phagocytosis or antigen uptake rather than active infection in order to avoid over-interpretation.

Thank you; we have clarified this point more explicitly in the revised version of our manuscript.

Along similar lines, a brief sentence noting that RNAscope analyses were exploratory and performed on a limited number of selected samples would better contextualize those results, and it would also be helpful to explicitly state that detection does not imply biological activity, replication competence, or fetal clinical impact.

Due to the exploratory approach with only nine samples, the interpretation of our results should be considered cautiously. We have made it very clear in the manuscript to prevent misunderstandings.

Reviewer #4:

I consider that Dr. Bartman and collaborators have a treasure trove of biological samples (placentas); however, the data they present leaves much to be desired, because in the immunohistochemistry, of three placentas positive for Nucleocapsid, if I understand correctly there is a signal for both viral antigens (Spike and N); however, there is a lot of positive signal for the Spike protein and very little signal for the Nucleocapsid protein, which contrasts with several articles, where a similar signal for both antigens has been reported. (They could discuss this part).

Thank you for pointing that out. When staining with spike and nucleocapsid antibodies, it is important to differentiate between the protein expression by vaccination effect, infection and the lingering effects of an infection. This is already part of our ‘Discussion’ section. For clearer understanding, we have discussed this point in detail.

Another core aspect of their work is their RNA scope, where they do not show a negative control, as apparently very little signal is observed in the positive samples, which leads me to think it could be a false positive.

Thank you for your important advice. Negative controls were already demonstrated in Figure 6. We have added the information of negative controls to the main manuscript.

Figure A, B: Placental specimen of a woman vaccinated by Comirnaty before and during pregnancy and with COVID-19 in the 36th week of pregnancy.

Figure 6A showed the positive result (BNT162b2-C1), Figure 6B the negative control (sensors V-nCOV2019-S Wuhan).

Figure C, D: Placental specimen of a woman vaccinated by Spikevax before pregnancy.

Figure 6C showed the positive result (S-encoding-mRNA-1273), Figure 6B the negative control (sensors V-nCOV2019-S Wuhan).

Finally, the authors mention that their work is novel because it is the first time traces of vaccine RNA in the placenta are reported, which is false, as it has already been reported by Xinhua. Lin, and published in JUNE of 2024, in American Journal of Obstetrics & Gynecology e115 (Transplacental transmission of the COVID-19 vaccine messenger RNA: evidence from placental, maternal, and cord blood analyses postvaccination). In this sense, what would be new about the work???

Many thanks for your comment. We have included both papers in our discussion and corrected the facts.

Finally, in Figure 6, they do not mention what the circles or the arrows represent.

Thank you for your advice. We have added it.
